# p53, estrogen and progesterone receptors in diagnostic curettage for endometrial adenocarcinoma and their correlation with morphological data and disease stage at hysterectomy

**DOI:** 10.1590/S1516-31802003000400005

**Published:** 2003-07-01

**Authors:** Vera Lúcia Leite Bonfitto, Liliana Aparecida Lucci de Angelo Andrade

**Keywords:** Endometrial carcinoma, p53, Estrogen receptor, Progesterone receptor, Carcinoma endometrial, p53, Receptor de estrógeno, Receptor de progesterona

## Abstract

**CONTEXT:**

Diagnostic staging is an important prognostic factor for endometrial adenocarcinoma. Apart from the histological type and histological grade, some markers seem to be associated with the stage and biological behavior of the disease. Among these are p53, estrogen and progesterone receptors.

**OBJECTIVE:**

The objectives of the present study were: to compare histological type and grading of endometrial carcinoma in curettage and hysterectomy samples; to assess expression of p53, estrogen and progesterone receptors in curettage specimens; and to correlate these data with morphology and staging of the disease in hysterectomy specimens.

**TYPE OF STUDY:**

Retrospective.

**SETTING:**

Department of Pathology, Faculdade de Ciências Médicas, Universidade Estadual de Campinas.

**SAMPLE:**

Histological diagnosis from 51 consecutive files.

**PROCEDURES:**

Immunohistochemical reactions for p53, estrogen and progesterone receptors via the avidinbiotin-peroxidase method in 51 curettage samples endometrial carcinoma were compared with the morphological data and disease stage in hysterectomy. Marker expression was correlated with histological type and grade and the final stage of the disease.

**RESULTS: According to the histological type:**

44 cases (86%) were of endometrioid and 7 (14%) non-endometrioid carcinoma. p53 expression was observed in 16% of endometrioid and 71% of non-endometrioid cases (p < 0.05). Although estrogen expression was more evident in endometrioid (54%) than non-endometrioid cases (29%), this was not statistically significant. Progesterone receptor expression was significantly higher in endometrioid than non-endometrioid cases (70% vs. 14%, p < 0.05). **According to the histological grade:** Estrogen and progesterone receptors were expressed more frequently in grade I endometrioid carcinoma, while p53 was mainly reported in tumor grades II and III. **According to final disease stage:** p53 and estrogen expression in curettage specimens was not related to stage; progesterone receptors, however, were expressed significantly less in advanced disease.

**CONCLUSION:**

p53 was observed in the majority of nonendometrioid and in high-grade endometrioid carcinoma, but was not related to stage. Estrogen and progesterone receptors were mainly found in grade I endometrioid carcinoma. The markers studied in curettage were no more valuable for predicting the disease stage than classical histological criteria.

## RESUMO

**CONTEXTO:**

O estádio do diagnóstico é importante fator prognóstico no carcinoma do endométrio. Além do tipo histológico e do grau de diferenciação da neoplasia, alguns marcadores parecem estar associados ao estádio e comportamento biológico da doença, entre eles, o p53 e os receptores de estrógeno e de progesterona.

**OBJETIVO:**

O objetivo foi comparar a expressão do p53 e os receptores de estrógeno e progesterona no material de curetagem diagnóstica para carcinoma, relacionando-os com o tipo e grau de diferenciação histológicos e com o estádio final da doença avaliado após em histerectomias.

**TIPO DE ESTUDO:**

Retrospectivo.

**LOCAL:**

Departamento de Anatomia Patológica/Faculdade de Ciências Médicas/Universidade Estadual de Campinas.

**PARTICIPANTES (AMOSTRA):**

Levantamento de diagnósticos histológicos de 51 casos consectivos do arquivo.

**PROCEDIMENTOS:**

51 curetagens de carcinoma foram estudadas através da reação imunoistoquímica para os marcadores p53, receptores de estrógeno e progesterona pelo método da avidina-biotina-peroxidase e os resultados foram comparados aos dados de histerectomia e estádio final da doença.

**VARIÁVEIS ESTUDADAS:**

Relação da expressão dos marcadores com o tipo e grau histológicos e o estádio final da doença.

**RESULTADOS:**

**Quanto ao tipo histológico:** 44 casos (86%) eram do tipo endometrióide e 7 (14%), não-endometrióide. A expressão do p53 foi observada em 16% dos carcinomas endometrióides e em 71% dos nãoendometrióides (p < 0,05). Embora a expressão do receptor de estrógeno fosse mais evidente nos endometrióides (54%) do que nos não-endometrióides (29%), isto não foi estatisticamente significativo. A expressão do receptor de progesterona foi significativamente maior no carcinoma endometrióide (70% X 14%, p < 0,05). **Quanto ao grau histológico:** o carcinoma endometrióide grau I expressou melhor os receptores de estrógeno e progesterona, enquanto p53 foi demonstrado principalmente nos tumores de graus II e III. **Estádio da doença na peça de histerectomia:** o p53 e o receptor de estrógeno na curetagem não foram relacionados com o estádio da doença. Já a expressão do receptor de progesterona foi significantemente menor nos estádios avançados.

**CONCLUSÃO:**

O p53 foi observado na maioria dos carcinomas não-endometrióides e nos endometrióides de alto grau, não estando relacionado com o estádio da doença. Os receptores de estrógeno e de progesterona são encontrados principalmente nos carcinomas endometrióides G I. Os marcadores estudados não superaram os critérios histológicos clássicos para predizer o estádio da doença.

## INTRODUCTION

Endometrial adenocarcinoma represents 90% of tumors in the uterine body and is the fourth commonest neoplasm in Western women and the fifth among Brazilians. Among gynecological cancers it ranks second as the cause of death.^1-3^ The incidence of this type of cancer has been increasing in the Brazilian population.^4^

The classical prognostic factors are histological type and grading, depth of myometrial invasion and extent in the cervix.^5-7^ It has been suggested that the expression of p53 protein, as well as estrogen and progesterone receptors, may have prognostic significance.^8,9^ p53 protein is expressed more in aggressive tumors, which suggests that mutation of the gene renders the neoplastic cells resistant to apoptosis.^10^ On the other hand, estrogen and progesterone receptors are usually associated with well-differentiated endometrial tumors.^8,10-12^

Our aims were: 1) to compare histological type and grading of endometrial carcinoma in curettage and hysterectomy samples; 2) to assess the expression of p53, estrogen and progesterone receptors in curettage specimens; and 3) to correlate these data with morphology and staging of the disease in the hysterectomy specimens.

## METHODS

A review was made of 51 consecutive cases of endometrioid carcinoma from January 1994 to December 1998. For all patients, diagnostic curettage and hysterectomy specimens, intraoperative frozen sections and paraffin sections, were available.

Representative 4 µm sections from for-malin-fixed, paraffin-embedded curettage specimens were histologically classified, according to the World Health Organization guidelines,^13^ as endometrioid or non-endometrioid types. Based on the International Federation of Gynecology and Obstetrics (FIGO) criteria,^14^ the histological grades for endometrial carcinoma are: well-differentiated or grade I; moderately differentiated or grade II; and poorly differentiated or grade III. In accordance with the disease stage, the histological parameters evaluated were depth of myometrial invasion, extent in the cervix and lymph node metastasis.

Further sections from the same tissue were submitted to immunohistochemical reactions for p53, estrogen and progesterone receptors, via the avidin-biotin-peroxidase method, using antigen recovery with microwave oven (15 minutes, citrate buffer 0.01N, pH 6.0). The primary antibodies for detecting p53 and progesterone receptors were, respectively: Dako D07 (code M7001), dilution 1:100; and Dako 1AE (code M3529-1), dilution 1:100. For estrogen, non-commercial antibody from a private source (University of Toulouse), was used at dilution 1:2000. Positive and negative controls for all procedures were obtained from duct breast carcinoma for estrogen and progesterone receptors and colon adenocarcinoma for p53. Immunohistochemical reactions occur in the nucleus, which acquires a brownish color; when more than 10% of tumor cell nuclei became stained, the reaction was considered positive.

The Fisher Exact test was used for statistical analysis of the positivity of the tests, in relation to histological type and grade and disease stage. The association was considered significant when p < 0.05.

## RESULTS

The sample consisted of 51 women with endometrioid carcinoma, with ages ranging from 43 to 83 years (mean of 64 years); 44 cases (86%) were of endometrioid and 7 (14%) nonendometrioid carcinoma (Figures 1 and 2). The 44 endometrioid carcinoma cases were graded as follows: grade I = 26/44 (59.1%), grade II = 13/44 (29.5%) and grade III = 5/44 (11.4%).

The curettage diagnoses were compared to those from the hysterectomy. Diagnostic agreement regarding histological type and grade was observed in 80%.

According to the stage of the disease the majority of the patients were diagnosed in stage I. However, non-endometrioid carcinoma was mainly diagnosed in more advanced stages (71%, Table 1).

**Table 1:** Stages and histological types of endometrial carcinoma specimens

	EC	(%)	NEC	(%)	TOTAL
S I	27/44	(61)	2/7	(29)	29
S II, III, IV	17/44	(39)	5/7	(71)	22
**TOTAL**	**44**		**7**		**51**

*S I, II, III, IV: stages I, II, III, IV; EC: endometrioid carcinoma; NEC: non-endometrioid carcinoma; (p = 0.216).*

**Markers and histological types:** p53 expression was found in 16% of the endometrioid and in 71% of the non-endometrial carcinomas (Figure 3); the estrogen and progesterone receptors were more frequent in the endometrioid type (Table 2).

**Table 2:** p53, estrogen and progesterone receptor expression and histological types of endometrial carcinoma specimens

Markers	EC	(%)	NEC	(%)	p
**p53**	7/44	(16)	5/7	71)	0.005
**ER**	24/44	(54)	2/7	(29)	0.248
**PR**	31/44	(70)	1/7	(14)	0.007

*EC: endometrioid carcinoma; NEC: non-endometrioid carcinoma; ER: estrogen receptor*;

*PR: progesterone receptor; p: significance by Fisher Exact test.*

**Markers and histological grade:** In the 44 cases of endometrioid carcinoma, those with grade I expressed estrogen (15 out of 26 cases = 63%) and progesterone receptors (21 out of 26 cases = 68%) more frequently than those with grades II and III. p53 was expressed in only 16% of endometrioid carcinomas, the majority of which were grade II or III (Table 3).

**Table 3:** p53, estrogen and progesterone receptor expression and histological grades of endometrioid carcinoma

Markers	EC G I	EC G II	EC G III	total (%)		p
**p53**	2/26	3/13	2/5	7/44	(16)	0.103
**ER**	15/26	6/13	3/5	24/44	(54)	0.829
**PR**	21/26	6/13	4/5	31/44	(70)	0.079

*EC G I, G II, G III: endometrioid carcinoma grades I, II and III; ER: estrogen receptor; PR: progesterone receptor; p: significance by Fisher Exact test.*

**Markers and final disease stage:** The expression of the markers in curettage material did not bear a significant relationship to the final disease stage as evaluated in the hysterectomy specimens (Table 4). We observed a slight increase in p53 positivity in the advanced stages.

**Table 4:** p53, estrogen and progesterone receptor expression and endometrial carcinoma stages

Markers	S I	(%)	S II, III, IV	(%)	total	(%)	p
**p53**	5/29	(17)	6/22	(27)	11/51	(22)	0.497
**ER**	15/29	(51)	11/22	(50)	26/51	(51)	1.000
**PR**	19/29	(65)	13/22	(59)	32/51	(63)	0.771

*S I, II, III, IV: stages I, II, III, IV; ER: estrogen receptor; PR: progesterone receptor; p: significance by Fisher Exact test.*

## DISCUSSION

Our results agree with the literature with regard to the higher frequencies of the endometrioid type and histological grade I (well-differentiated) carcinomas.^5-7,15-17^ Non-endometrioid carcinoma predominated in elderly women, as expected, although the frequency of 14% was higher than found in other studies.^5,6^ This difference may be attributable to the relatively small size of the population studied and the fact that our service is a reference center in which complex cases are concentrated.

The 80% diagnostic agreement between the two procedures can be explained by the small size of the fragments obtained at curettage. Most patients were in stage I at diagnosis: this correlates with the predominance of the endometrioid type, which has slower progression. On the other hand, most non-endometrioid carcinomas were in stages II or III, although this was not statistically significant due to the small size of the sample. All these observations are in accordance with the literature.^5,6,15,18^

p53 was significantly more expressed in non-endometrioid (71%) than in endometrioid carcinoma (16%), and was mostly in histological grades II and III. This indicates possible mutation of p53 as an early event, which is typical of the carcinogenic pathway for non-endometrioid carcinomas.^19-21^ Conversely, estrogen and progesterone receptors are more frequently positive in endometrioid carcinoma, particularly if well-differentiated, as reported by others.^9,18,21-23^ However, in our results, only progesterone receptor expression was statistically significant, which could possibly be explained by the antibodies coming from different sources or having different sensitivity and specificity, as reported by others.^23^

We were able to confirm that p53 expression is inversely related to that of estrogen and progesterone receptors, thus indicating a dual theory of carcinogenesis in the endometrium.^11,23-26^

Expression of the markers studied was not significantly associated with the disease stage. It has been reported that p53 expression correlates with more advanced stages.^23,25,27,28^ Although we found slightly greater p53 expression in stages II and III, this was not statistically significant.

## CONCLUSION

We conclude that the expression of p53, estrogen and progesterone receptors in curettage material was no more valuable for predicting the disease stage than the classical morphological criteria of histological type and grading.

## References

[1] Ball HG, Blessing JA, Lentz SS, Mutch DG (1996). A phase II trial of paclitaxel in patients with advanced or recurrent adenocarcinoma of the endometrium: a Gynecologic Oncology Group study. Gynecol Oncol.

[2] Parker SL, Tong T, Bolden S, Wingo PA (1996). Cancer statistics, 1996. CA Cancer J Clin.

[3] Brasil (1993). Ministério da Saúde, Instituto Nacional do Câncer/Inca, Coordenação de Programas de Controle do Câncer. [Coordination Office for Cancer Control Programs]. Pró-câncer no Brasil: dados dos registros de base hospitalar.

[4] Pedro AO, Pinto-Neto AM, Costa-Paiva LHS, Borges MH, Lane E (1996). A neoplasia maligna como causa de óbito feminino no Complexo Hospitalar da Unicamp: um aspecto de transição epidemiológica. Rev Bras Ginecol Obstet.

[5] Kurman RJ, Zaino RJ, Norris HJ, Kurman RJ (1994). Endometrial carcinoma. Blaustein's pathology of the female genital tract.

[6] Gompel C, Silverberg SG, Gompel C, Silverberg SG (1994). The Corpus Uteri. Pathology in Gynecology and Obstetrics.

[7] Hendrickson MR, Kempson RL, Sternberg SS, Antonioli DA, Mills SE, Carter D, Oberman HA (1994). The Uterine Corpus. Diagnostic Surgical Pathology.

[8] Berchuck A, Kohler MF, Marks JR, Wiseman R, Boyd J, Bast RC (1994). The p53 tumor suppressor gene frequently is altered in gynecologic cancers. Am J Obstet Gynecol.

[9] Geisler JP, Wiemann MC, Zhou Z, Miller GA, Geisler HE (1996). p53 as a prognostic indicator in endometrial cancer. Gynecol Oncol.

[10] Burton JL, Stewart RL, Healtley MK, Royds JA, Wells M (1999). p53 expression, p21 expression and the apoptotic index in endometrial endometrial adenocarcinoma. Histopathology.

[11] Bokhman JV (1983). Two pathogenetic types of endometrial carcinoma. Gynecol Oncol.

[12] Honda T, Kato H, Imamura T (1993). Involvement of p53 gene mutations in human endometrial carcinomas. Int J Cancer.

[13] Scully RE, Bonfiglio TA, Kurman RJ, Silverberg SG, Wilkinson EJ (1994). Histological Typing of Female Genital Tract Tumours: in collaboration with Pathologists in 10 Countries.

[14] Shepherd JH (1989). Revised FIGO staging for gynaecological cancer. Br J Obstet Gynaecol.

[15] Burton JL, Wells M (1998). Recent advances in the histopathology and molecular pathology of carcinoma of the endometrium. Histopathology.

[16] Ioffe OB, Papadimitriou JC, Drachenberg CB (1998). Correlation of proliferation indices, apoptosis, and related oncogene expression (bcl-2 and c-erbB-2) and p53 in proliferative, hyperplastic, and malignant endometrium. Hum Pathol.

[17] Noumoff JS, Menzin A, Mikuta J (1991). The ability to evaluate prognostic variables on frozen section in hysterectomies performed for endometrial carcinoma. Gynecol Oncol.

[18] Kounelis S, Kapranos N, Kouri E, Coppola D, Papadaki H, Jones MW (2000). Immunohistochemical profile of endometrial adenocarcinoma: a study of 61 cases and review of the literature. Mod Pathol.

[19] Mutter GL (2000). Endometrial intraepithelial neoplasia (EIN): will it bring order to chaos? The Endometrial Collaborative Group. Gynecol Oncol.

[20] Lax SF, Kendall B, Tashiro H, Slebos RJ, Hedrick L (2000). The frequency of p53, K-ras mutations, and microsatellite instability differs in uterine endometrial and serous carcinoma evidence of distinct molecular genetic pathway. Cancer.

[21] Fernando SSE, Wu X, Perera LS (2000). p53 Overexpression and Steroid Hormone Receptor Status in Endometrial Carcinoma. Int J Surg Pathol.

[22] Hamel NW, Sebo TJ, Wilson TO (1996). Prognostic value of p53 and proliferating cell nuclear antigen expression in endometrial carcinoma. Gynecol Oncol.

[23] Ito K, Watanabe K, Nasim S (1994). Prognostic significance of p53 overexpression in endometrial cancer. Cancer Res.

[24] Taskin M, Lallas TA, Barber HR, Shevchuk MM (1997). bcl-2 and p53 in endometrial adenocarcinoma. Mod Pathol.

[25] Ozsaran AA, Tüker S, Dikmen Y (1999). p53 staining as a prognostic indicator in endometrial carcinoma. Eur J Gynaecol Oncol.

[26] Elhafey AS, Papadimitriou JC, El-Hakim MS (2001). Computerized image analysis of p53 and proliferating cell nuclear antigen expression in benign, hyperplastic, and malignant endometrium. Arch Pathol Lab Med.

[27] Ambros RA, Vigna PA, Figge J (1994). Observations on tumor and metastatic suppressor gene status in endometrial carcinoma with particular emphasis on p53. Cancer.

[28] Rose PG (1996). Endometrial carcinoma. N Engl J Med.

